# Membrane proximal ectodomain cleavage of MUC16 occurs in the acidifying Golgi/post-Golgi compartments

**DOI:** 10.1038/srep09759

**Published:** 2015-06-05

**Authors:** Srustidhar Das, Prabin D. Majhi, Mona H. Al-Mugotir, Satyanarayana Rachagani, Paul Sorgen, Surinder K. Batra

**Affiliations:** 1 Department of Biochemistry and Molecular Biology, University of Nebraska Medical Center, Omaha, NE 68198, USA; 2 Department of Pathology, University of Nebraska Medical Center, Omaha, NE 68198, USA; 3 Buffett Cancer Center, Eppley Institute for Research in Cancer and Allied Diseases, University of Nebraska Medical Center, Omaha, NE 68198, USA

## Abstract

MUC16, precursor of the most widely used ovarian cancer biomarker CA125, is up regulated in multiple malignancies and is associated with poor prognosis. While the pro-tumorigenic and metastatic roles of MUC16 are ascribed to the cell-associated carboxyl-terminal MUC16 (MUC16-Cter), the exact biochemical nature of MUC16 cleavage generating MUC16-Cter has remained unknown. Using different lengths of dual-epitope (N-terminal FLAG- and C-terminal HA-Tag) tagged C-terminal MUC16 fragments, we demonstrate that MUC16 cleavage takes place in the juxta-membrane ectodomain stretch of twelve amino acids that generates a ~17 kDa cleaved product and is distinct from the predicted sites. This was further corroborated by domain swapping experiment. Further, the cleavage of MUC16 was found to take place in the Golgi/post-Golgi compartments and is dependent on the acidic pH in the secretory pathway. A similar pattern of ~17 kDa cleaved MUC16 was observed in multiple cell types eliminating the possibility of cell type specific phenomenon. MUC16-Cter translocates to the nucleus in a cleavage dependent manner and binds to the chromatin suggesting its involvement in regulation of gene expression. Taken together, we demonstrate for the first time the oft-predicted cleavage of MUC16 that is critical in designing successful therapeutic interventions based on MUC16.

Mucins are high molecular weight glycoproteins and are primarily expressed by the secretory epithelial cells lining respiratory, gastrointestinal and reproductive tracts^1^. Although mucins are thought to protect epithelial surfaces from various physical insults, recent molecular studies have generated interest in their use as diagnostic and therapeutic targets^1^,^2^,^3^ for their role in cancer. In particular, the transmembrane mucins have been implicated in various oncogenic signaling pathways and their *de novo* expression in certain malignancies renders them very attractive targets^1^,^2^,^3^. MUC16 (CA125), the best-known biomarker for ovarian cancer^4^, is up regulated in multiple malignancies and is strongly associated with poor prognosis^5^,^6^,^7^. MUC16 is a type-I transmembrane protein with a heavily glycosylated N-terminal region, a tandem repeat region comprising of approximately 60 repeats of ~156 amino acids each, a transmembrane (TM) domain and a cytoplasmic tail domain (CTD) of 32 amino acids^8^,^9^. MUC16 is predicted to harbor ~56 SEA (Sperm protein, Enterokinase and Agrin) domains unlike other mucins such as MUC1, MUC12, MUC13 and MUC17 which possess only a single SEA module^9^,^10^.

SEA domain(s) present in mucin and non-mucin proteins are shown to possess autoproteolytic activity^11^,^12^,^13^. MUC16 is speculated to harbor two proteolytic sites in the membrane proximal SEA domains i.e. 50 residues proximal to the TM domain (site #1 at PLARRVDR) in the last (56^th^)^14^ and the ‘DSVLV’ site (site #2) analogous to the MUC1 ‘GSVVV’ site in the penultimate (55^th^) SEA domains^12^. However, neither has been experimentally validated. There have been studies addressing the functional significance of various lengths of MUC16 carboxyl-terminal region (283 and 413 amino acids) in ovarian, breast and colon cancer cells, however, the exact biochemical nature of MUC16 cleavage was not addressed in these studies^15^,^16^,^17^,^18^. Besides, while the autoproteolytic cleavage in the last and penultimate SEA domain has been hypothesized to be the major proteolytic mechanism of MUC16 cleavage, proteases such as MMP-7, neutrophil elastase (NE) and bacterial metalloprotease (ZmpC) have been implicated in enhanced shedding of MUC16 from the cell surface^19^,^20^.

Given the importance of CA125 in ovarian cancer, antibodies such as Oregovomab and Abagovomab against CA125 have been used in clinical trials for ovarian cancer patients without positive outcomes^21^,^22^,^23^. Since these antibodies bind to the extracellular portion of MUC16 (i.e. CA125), the potential reasons for the failure of these antibodies are (i) binding of these antibodies to the circulating (shed) CA125, therefore, reducing the amount of antibodies available to target and kill the cancer cells, (ii) the kinetics and dynamics of MUC16 cleavage/shedding from the tumor cells is not well understood, therefore, the likelihood of shedding of cell-surface MUC16 would still reduce the availability of these therapeutic antibodies to the cancer cells^21^. Taken together, an understanding of biochemical nature of MUC16 cleavage and its potential regulators would be critical in devising successful therapeutic strategy based on MUC16/CA125.

For the first time, we report here the experimental evidence for the oft-predicted cleavage of MUC16. This takes place in the juxta membrane ectodomain region and is distinct from the predicted sites. The cleavage is independent of extracellular proteases (i.e. NE and MMP-7) and intracellular cues (i.e. phosphorylation). Further, the cellular location of cleavage is identified to be the Golgi/post-Golgi compartments and MUC16 cleavage is dependent on the acidic pH in the secretory pathway. MUC16-Cter translocates to the nucleus in a cleavage dependent manner and independent of the putative nuclear localization signal (NLS) and participates in the regulation of gene expression^24^. Altogether, the present study provides insight into the cleavage of MUC16 that is critical towards understanding its functional significance under physiological and pathological conditions and subsequent therapeutic targeting in multiple cancer types.

## Results

### Membrane proximal ectodomain cleavage of MUC16 is spatially distinct from the predicted cleavage sites

**Figure 1:**
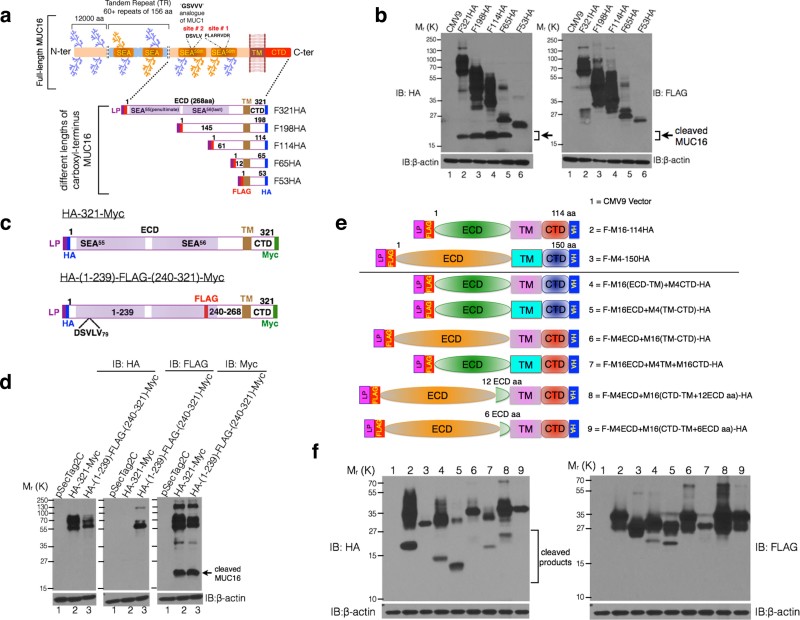
Membrane proximal ectodomain cleavage of MUC16. (a) Schematic representation of full-length and different lengths of MUC16-Cter fragments with N-terminal FLAG and C-terminal HA-tag cloned into the p3X-FLAG-CMV9 vector (CMV9) with a preprotrypsin leader peptide (LP). The predicted cleavage sites in the last (site #1, PLARRVDR) and penultimate (site #2, DSVLV) SEA domains are indicated. (b) HEK293T cells were transiently transfected with the plasmids mentioned in (a) and were immunoblotted with anti-FLAG and anti-HA antibodies. Cleaved MUC16 is indicated by an arrow in the HA immunoblot. (c and d) Multiple cleavage events were not observed in the MUC16 carboxyl terminal region as predicted. (c) Schematic representation of a 321 amino acids fragment of the MUC16-Cter region cloned into the pSecTag2C vector with an Ig-κ leader peptide. N-terminal HA and C-terminal Myc-tags were added, with or without an internal FLAG-tag to identify multiple cleavage sites upstream with particular emphasis on the predicted DSVLV site in the penultimate SEA domain. (d) HEK293T cells were transfected with the plasmids mentioned in (c) and were immunoblotted with anti-HA, FLAG and Myc antibodies. (e and f) Domain swapping experiment reiterates the cleavage of MUC16 in the membrane proximal 12 amino acids. (e) Schematic representation of various domains of 114 and 150 amino acids from the C-ter fragments of MUC16 and MUC4 respectively (i.e. ECD, TM and CTD as shown in the schematic, top panel) were swapped with each other (bottom panel of the schematics) and cloned into the CMV9 vector with N-terminal FLAG and C-terminal HA tags. (f) HEK293T cells were transiently transfected with the plasmids shown in (e). Cell lysates were immunoblotted with anti-FLAG and anti-HA antibodies to assess the effect of different domains on cleavage.

Cleavage of MUC16 has been proposed to take place in the DSVLV site of penultimate SEA domain, analogous to MUC1 cleavage site GSVVV and/or at 50 residues proximal to transmembrane (TM) domain in the last SEA domain^12^,^14^. However, neither has been experimentally validated. Due to lack of antibodies for the juxta-membrane region of MUC16, we demonstrated cleavage of MUC16 using dual-epitope tagging (Fig. 1a and 1b). We generated a mammalian expression construct with last two SEA domains of MUC16 (321 amino acids from the C-terminal end, termed as F321HA) that included both the predicted cleavage sites and has N-terminal preprotrypsin leader peptide for appropriate membrane targeting. Further, multiple deletion constructs were generated from F321HA (Fig. 1a). Expression of different lengths of dual-tagged MUC16 carboxyl-terminus (MUC16-Cter) in HEK293T, except the F53HA lacking any extracellular residues, resulted in a unique ~17 kDa product present in HA but not FLAG immunoblot (indicated by an arrow in Fig. 1b). This demonstrates that MUC16 undergoes cleavage in the carboxyl-terminal region as the C-terminal (HA-tagged) product is physically separated from the N-terminal portion (FLAG-tagged) resulting in a unique ~17 kDa fragment. Our findings demonstrate that C-terminal 65 residues of MUC16 (F65HA) is the minimal length that is capable of undergoing cleavage, which harbors only 12 extracellular amino acids in addition to the transmembrane (TM) and cytoplasmic tail domain (CTD) and no SEA domain. However, this does not rule out further cleavage(s) upstream in the last and in the penultimate SEA domains. To assess additional cleavage(s) of MUC16 in addition to the afore-mentioned cleavage, a triple epitope-tagged construct was engineered wherein a FLAG-tag was introduced between the N-terminal HA-tag and C-terminal Myc-tag at 29^th^ membrane-proximal ectodomain residue into the 321 amino acids fragment of MUC16 (Fig. 1c). Further upstream cleavage(s) in addition to the cleavage at the juxtamembrane ectodomain would result in unequal sized HA- and FLAG-tagged products. However, the size of the FLAG-tagged product was same as the corresponding HA-tagged product, suggesting no additional cleavage further upstream (Fig. 1d). For the first time, we present experimental evidence demonstrating cleavage of MUC16 and that the site of cleavage is distinct from the predicted cleavage sites^12^,^14^.

To further substantiate our findings, we swapped various domains (extracellular domain (ECD), TM and CTD) of 150 amino acid fragment of another transmembrane mucin MUC4 with that of 114 amino acid fragment of MUC16 (Fig. 1e). MUC4 is a non-SEA domain mucin with a short CTD (20 amino acids) and F-M4-150HA does not undergo cleavage (Fig. 1f, lane 3) unlike that of 114 amino acid fragment of MUC16 (Fig. 1f, lane 2) and therefore was considered to be an ideal partner for swapping. Swapping the TM or CTD alone or together (TM-CTD) of MUC4 with that of MUC16 did not prevent its cleavage (Fig. 1f, lanes 4, 5 and 7). However, replacing the extracellular domain (ECD) of MUC16 with MUC4 abrogated its cleavage (Fig. 1f, lane 6) that was partially rescued by inclusion of twelve (Fig. 1f, lane 8), but not six (Fig. 1f, lane 9) membrane-proximal residues of MUC16. These findings further corroborate membrane proximal ectodomain cleavage of MUC16 and that the 12 extracellular residues are critical for this cleavage.

### N-glycosylation and ubiquitylation of carboxy-terminus of MUC16 determine its size and stability

**Figure 2:**
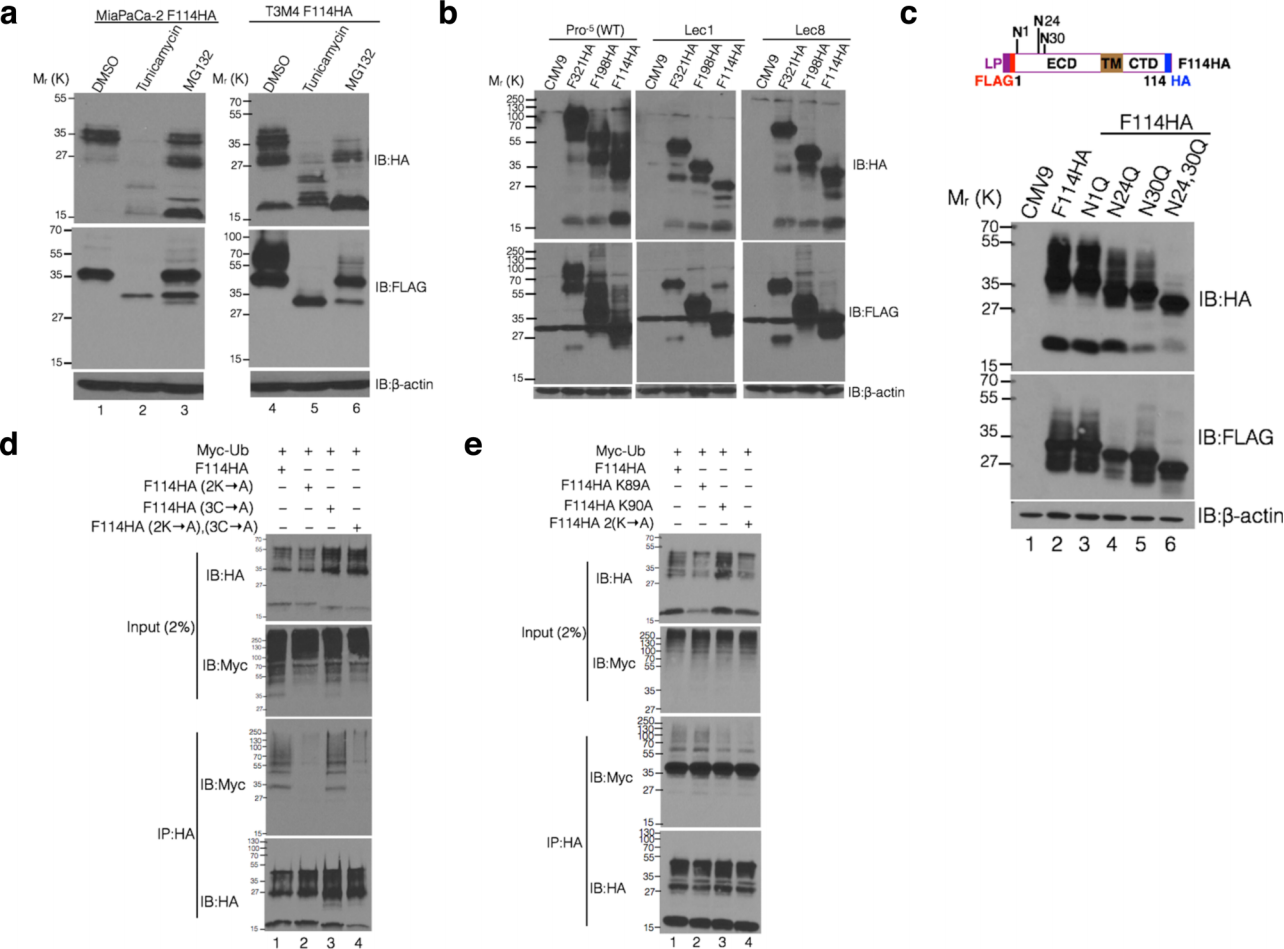
MUC16-Cter undergoes N-glycosylation and ubiquitylation that determine its stability. (a) MiaPaCa-2 and T3M4 PC cells stably transfected with F114HA were treated with either Tunicamycin (5 μg/ml) or MG132 (10 μM) for the indicated times and cell lysates were immunoblotted with indicated antibodies. (b) Wild type (Pro^−5^) and N-glycosylation defective (Lec1 and Lec8) Chinese Hamster Ovary cells were transiently transfected with MUC16-Cter constructs of various lengths and the cell lysates were immunoblotted with indicated antibodies. (c) Aspargines (N) that are predicted to be N-glycosylated (N-X-S/T, upper panel schematic) were mutated to glutamines (Q) either individually or in combination in the F114HA construct, and the mutated constructs were transiently transfected into HEK293T cells. Cell lysates were immunoblotted with indicated antibodies. (d) *In vivo* ubiquitylation was carried out by cotransfecting Myc-tagged ubiquitin with wild type F114HA (lane-1) or lysine (F114HA Lys89,90Ala (2K → A), lane-2) or cysteine (F114HA Cys76,79,100Ala, (3C → A), lane-3) or both lysine and cysteine (F114HA 2K → A, 3C → A, lane-4) mutants into HEK293T cells. HA-tagged MUC16-Cter was immunoprecipitated using anti-HA antibodies. Ubiquitylation was detected using anti-Myc antibodies. MUC16-Cter and ubiquitin were detected in the whole cell lysates using anti-HA and anti-Myc antibodies (Input). (e) Similar *in vivo* ubiquitylation was carried out by cotransfecting Myc-tagged ubiquitin with wild type F114HA (lane-1) or Lys89Ala (F114HA K89A, lane-2) or Lys90Ala (F114HA K90A, lane-3) or Lys89,90Ala (F114HA 2K → A, lane-4) mutants into HEK293T cells.

MUC16-Cter cleavage, studied with F114HA (a more detailed description including the amino acids composition of the 114 carboxyl-terminal residues of MUC16 is provided in Supplementary Fig. 1) and other constructs, yielded a unique 17 kDa HA-only product but also showed a number of products with both HA and FLAG-tags of molecular weight disproportionate to the polypeptide length (Fig. 1b). This suggests that (i) MUC16-Cter undergoes partial cleavage, leaving much of the protein uncleaved which undergoes various post-translational modifications and/or (ii) both ends of the cleaved protein remain associated, in specific (heterodimeric) or non-specific interactions (aggregation). Tunicamycin or MG132 treatment of MiaPaCa-2 and T3M4 pancreatic cancer cells stably transfected with CMV9-F114HA resulted significant abrogation of higher molecular weight forms and increased accumulation of MUC16-Cter, respectively (Fig. 2a) suggesting MUC16-Cter undergoes N-glycosylation and ubiquitylation. To assess the global contribution of N-glycosylation, Chinese Hamster Ovary (CHO) cells with intact (Pro^−5^) and defective N-glycosylation (Lec1 and Lec8)^25^ were transfected with different lengths of MUC16-Cter, which affected both the size and stability of the proteins (Fig. 2b). Further, mutation of three N-glycosylation sites (N-X-S/T) (Supplementary Fig. 1) in F114HA to Gln (Q) corroborated the above findings (Fig. 2c). To investigate whether MUC16-Cter undergoes ubiquitylation, *in vivo* ubiquitylation assay was performed using the wild type, Lys and Cys mutants. *In vivo* ubiquitylation of wild type, Lys (2K → A) and Cys (3C → A) mutants of MUC16-Cter (F114HA) showed that the Lys in the CTD (Supplementary Fig. 1) of MUC16 undergoes polyubiquitylation (Fig. 2d) and therefore proteasomal degradation (Fig. 2a). Similar ubiquitylation analysis using individual Lys mutations demonstrated that Lys90 is preferentially ubiquitylated than Lys89 (Fig. 2e). Taken together, while both N-glycosylation and ubiquitylation can influence the function of MUC16-cter by regulating its stability, the exact function(s) of site specific N-glycosylation and ubiquitylation remains to be elucidated.

### MUC16 cleavage is unaffected by proteases involved in regulated intramembrane proteolysis (RIP), neutrophil elastase or MMP-7 and is independent of intracellular cue(s)

**Figure 3:**
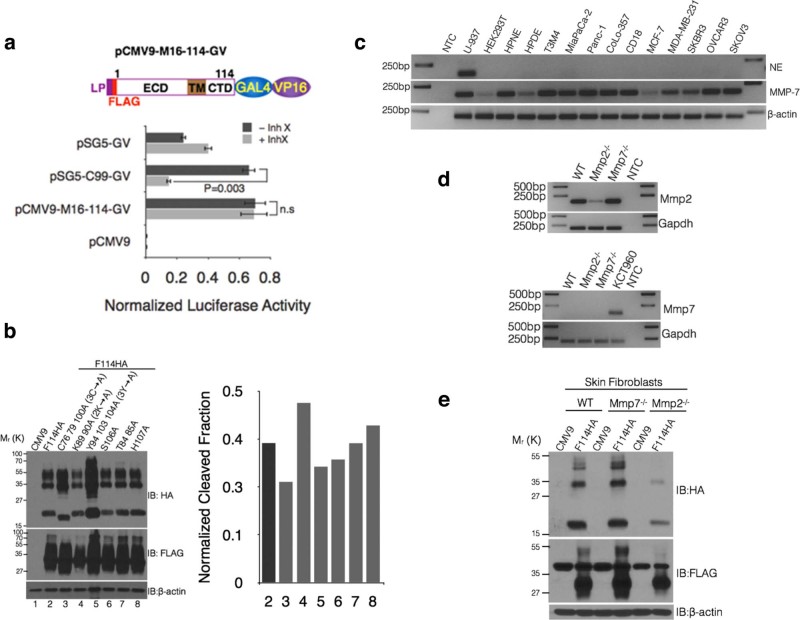
Cleavage of MUC16 is independent of Υ-secretase, neutrophil elastase and MMP-7 and intracellular cues. (a) MUC16 does not undergo Υ-secretase-mediated regulated intra-membrane proteolysis (RIP). Schematic representations of luciferase reporter construct to assess RIP of MUC16-Cter. A GAL4 (DNA-binding domain)-VP16 (Activation domain) fusion was cloned into the C-terminus of the MUC16-Cter (CMV9-FLAG-114 amino acid fragment) (top panel). Bottom panel: HEK293T cells were cotransfected with empty CMV9 vector (pCMV9) or with M16-114-GAL4-VP16 (pCMV9-M16-114-GV) or APP-C99-GAL4-VP16 (pSG5-C99-GV, positive control for Υ-secretase cleavage) and a luciferase reporter driven by the GAL4 upstream sequence (pFR-Luc) along with pRenilla-Luc for transfection control in the presence or absence of Υ-secretase inhibitor, Inhibitor X (Inh X). The bars represent the normalized luciferase activity of pFR-Luc to pRenilla-Luc of a representative experiment and is presented as mean ± s.e.m, n = 3. (b) MUC16 cleavage was independent of intracellular cues. Amino acids capable of any kind of post-translational modifications were mutated to Ala in the CMV9-F114HA construct and were transfected into HEK293T cells. Cell lysates were immunoblotted with qanti-HA and FLAG antibodies (left panel). Bars on the right represent the normalized cleaved fraction measured by generating a ratio of normalized (with actin) bottom-HA/total-HA (see cleaved fraction calculation in materials and methods). (c) Expression of *ELA2* and *MMP-7* in multiple cells lines. Expression of *ELA2* and *MMP-7* were assessed using reverse transcriptase PCR (RT-PCR). U-937 cells were used as a positive control for *ELA2* expression. (d) Skin fibroblasts established from Mmp7^−/−^ and Mmp2^−/−^ mice were analyzed for the expression of *Mmp2* and *Mmp7* using RT-PCR. KCT960 cells were used as a positive control for *Mmp7* expression. (e) Skin fibroblasts from Mmp7^−/−^ and Mmp2^−/−^ mice were transiently transfected with control (CMV9) and MUC16-Cter (F114HA) plasmids and the cell lysates were immunoblotted with respective antibodies to assess the role of Mmp7 (and therefore MMP7) in the cleavage of MUC16. Mmp2^−/−^ fibroblasts were used as a control.

Having demonstrated no upstream cleavage(s) in the last and penultimate SEA domains, we next explored the possibility of downstream cleavage site(s). Specifically, we investigated the regulated intramembrane proteolysis (RIP) by γ-secretase, since (i) exclusion of the extracellular 12 amino acids in F53HA that does not undergo cleavage, would have abrogated the cleavage by priming proteases (such as TACE for Notch1^26^ and α-secretase for APP^27^) and (ii) γ-secretase inhibitor treatment in HCLE and HCjE cells reduced MUC16 protein without affecting mRNA^28^. While the reduced MUC16 protein is attributed to Notch signaling^28^, a direct effect of γ-secretase on MUC16 cannot be ruled out. Using a quantitative luciferase assay based on the GAL4-VP16 system^29^, we showed that MUC16 is not a target of γ-secretase (Fig. 3a) where the amyloid precursor protein (APP-C99-GAL4-VP16) was used as a positive control. Although γ-secretase^30^ mediated RIP is the most widely studied regulated intramembrane proteolysis, other proteases such as site-2 proteases^30^,^31^ and rhomboid proteases^30^ have been shown to belong to the same clan. However, swapping the TM domain alone or TM-CTD together of MUC4 with MUC16 did not abrogate the cleavage of MUC16 ruling out the involvement of any form of RIP (Fig. 1f, lanes 5 and 7).

Previous studies have reported that Ser/Thr phosphorylation in the CTD of MUC16 (Supplementary Fig. 1) could be a trigger for proteolytic cleavage of MUC16 in response to EGF treatment^32^,^33^; however, mutating Ser106Ala or Thr84/85Ala did not affect the cleavage of MUC16-Cter (Fig. 3b, lanes 6 and 7). Further, amino acids capable of any post-translational modification(s) were mutated to Ala without any effect on cleavage (Fig. 3b right panel, *see cleaved fraction calculation in Methods*) and replacement of the entire CTD of MUC4 with that of MUC16 did not abrogate cleavage (Fig. 1f, lane 4). These results indicate no requirement of cytoplasmic cues for MUC16 cleavage. However, mutating Tyr94/103/104Ala (F114HA3Y → A) (Supplementary Fig. 1) resulted in an increase in both the unique HA-tagged and common FLAG and HA-tagged products (Fig. 3b, lane 5). Although it appears that Tyr mutations lead to increased accumulation of the cleaved MUC16, the ratio of cleaved (17 kDa HA-tagged product) to the total HA-tagged fraction (17 kDa + higher molecular weight HA-tagged products) remains unchanged (*see cleaved fraction calculation in Methods*). This suggests that Tyr phosphorylation leads to increased degradation (rapid turnover) of MUC16. A recent study^17^ has demonstrated that Tyr22142 (same as Tyr104 in our study) of MUC16 is phosphorylated by c-Src.

Without intracellular cues for MUC16 cleavage, we examined the involvement of proteases such as NE and MMP-7 as their treatment resulted in enhanced shedding of MUC16 in HCLE cells^19^. We analyzed many types of non-neutrophil cell lines for NE production, but none expressed NE (Fig. 3c). However, MUC16 cleavage was observed in all the cell types (Supplementary Fig. 2), therefore, ruling out the involvement of NE. In addition, the observation of a similar cleavage pattern in all the cell lines examined showed similar processing of MUC16 irrespective of cell types used, eliminating the possibility of cell type specific effect (Supplementary Fig. 2). This suggests that while MUC16 cleavage takes place in all the cell types, whether it will have similar phenotypes and mode of action need to be addressed in future studies. Although MMP-7 was expressed by all the cells (Fig. 3c), cleavage of MUC16 occurs in Mmp7^−/−^ skin fibroblasts (Fig. 3d and 3e), ruling out its involvement in MUC16 cleavage. Taken together, these results indicate that MUC16 is neither a target of previously reported extracellular proteases such as NE and MMP-7 nor intramembrane proteolysis and its cleavage is independent of post-translational modifications in the CTD.

### Cleavage of MUC16 takes place in the acidic pH of Golgi/post-Golgi compartments

**Figure 4:**
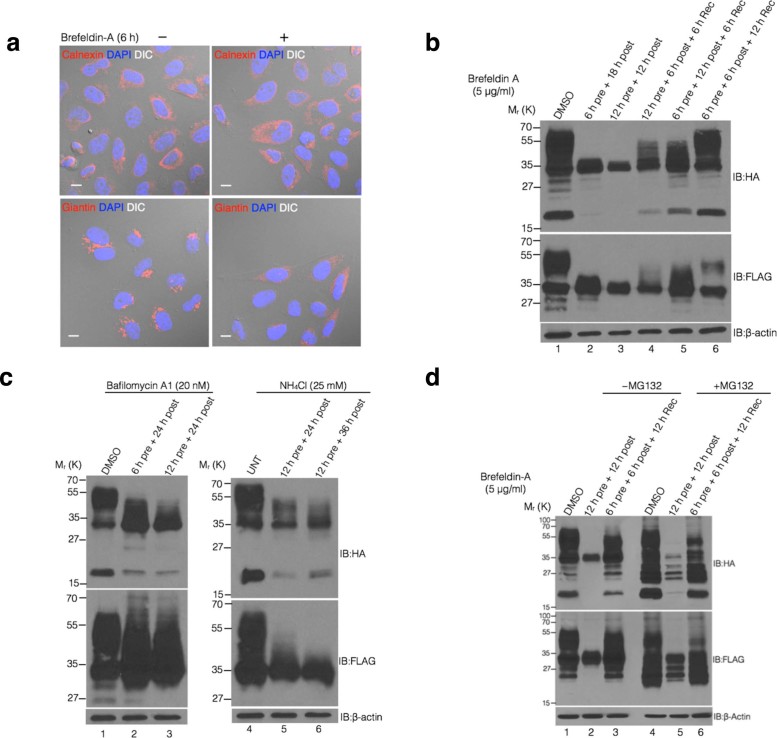
MUC16 cleavage takes place in the acidic pH of Golgi/post-Golgi compartments. (a) Dissolution of Golgi structures following Brefeldin-A (BFA) treatment. Immunofluorescence analysis of untreated or BFA treated (for 6 h) HeLa cells were performed using organelle marker antibodies for endoplasmic reticulum (Calnexin), Golgi (Giantin) and nucleus (DAPI). Scale bar, 10 μm. (b) HeLa cells pretreated with brefeldin-A (BFA, 5 μg/ml) were transfected with F114HA (CMV9-F114HA unless otherwise mentioned) while being maintained in BFA followed by post-treatment (lanes 2-6) and allowed to recover by removing BFA (lanes 4-6) for indicated times. Cell lysates were immunoblotted with indicated antibodies. (c) Intra-Golgi/post-Golgi pH is critical for cleavage of MUC16. HeLa cells pretreated with either bafilomycin-A1 (BafA1, 20 nM) or NH_4_Cl (25 mM) were transfected with F114HA while being maintained in BafA1 or NH_4_Cl followed by post-treatment for indicated times. Cell lysates were immunoblotted with indicated antibodies. (d) BFA treatment affects cleavage of MUC16-Cter not the degradation. HeLa cells pretreated with brefeldin-A (BFA, 5 μg/ml) were transfected with F114HA while being maintained in BFA followed by post-treatment and allowed to recover by removing BFA for indicated times. Twelve hours before collecting the lysates, the cells were either control treated (DMSO) or with 10 μM MG132. Cell lysates were immunoblotted with indicated antibodies.

Next, to find out the exact cellular location for cleavage, we used brefeldin-A (BFA), which induces fusion of Golgi to the endoplasmic reticulum (ER) (Fig. 4a), resulting in redistribution of Golgi proteins into the ER^34^ and a rapid and reversible block in the ER-Golgi trafficking that prevents further secretion^35^. Pretreatment of HeLa cells with BFA resulted in abrogation of MUC16-Cter cleavage (Fig. 4b, lanes 2–3) that was rescued by BFA removal (Fig. 4b, lanes 4–6). One major distinction between the ER and the Golgi/post-Golgi compartments is the existence of a pH gradient with values decreasing towards the secretory destination from the ER (pH ~ 7.1), cis-medial-trans Golgi (pH ~ 7.0–6.0) and endosomes (pH ~ 5.0)^36^. If MUC16 cleavage was induced at the acidic pH of the Golgi/post-Golgi compartments, we reasoned that alkalinization of the Golgi/post-Golgi compartments could abrogate cleavage. Towards this, we used two pH-disrupting agents: NH_4_Cl, a weak base, which neutralizes the acidic pH by releasing ammonia into the cell, and bafilomycin-A1, an inhibitor of the H^+^-K^+^-ATPase pumps present in both secretory and endocytic pathways^37^. HeLa cells pretreated with either NH_4_Cl or bafilomycin-A1 demonstrated that neutralizing the pH of the Golgi indeed significantly abrogated MUC16-Cter cleavage (Fig. 4c). To rule out degradation, we showed that MG132 treatment of BFA treated cells did not lead to accumulation of the cleaved fragment (Fig. 4d, lanes 2 and 5). Taken together, these results indicate that cleavage of MUC16 takes place in the acidic pH of the Golgi/post-Golgi compartments.

### Cleavage of MUC16 is not dictated by its primary amino acid sequence

**Figure 5:**
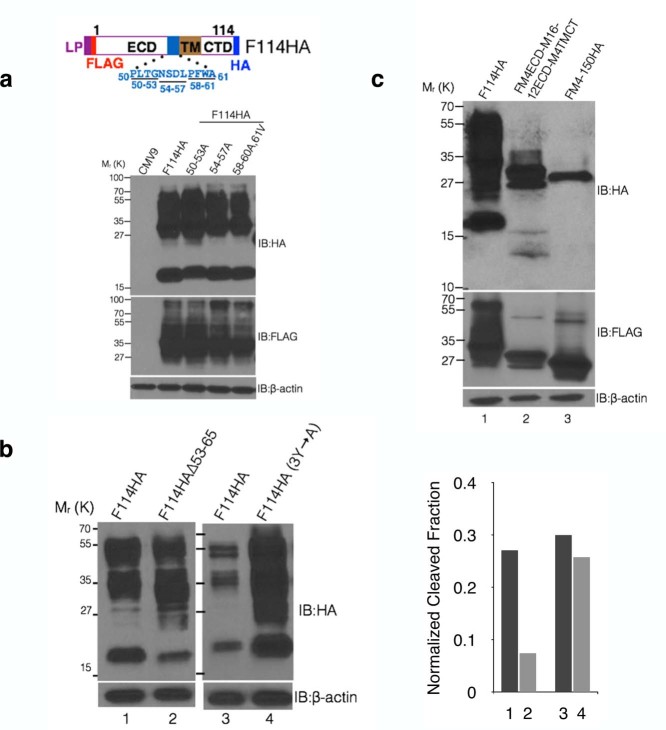
Cleavage of MUC16 is independent of its primary amino acid sequence. (a) Twelve membrane proximal residues of MUC16-Cter were mutated four at a time to alanine/valine in the F114HA construct (top panel). The resultant plasmids were transiently transfected into HEK293T cells and the cell lysates were immunoblotted with the indicated antibodies (bottom panel). (b) Plasmids encoding F114HA, F114HAΔ53-65 (deleted membrane-proximal 12 amino acids) and tyrosine (F114HA-Tyr94/103/104Ala i.e. Y3A) mutants were transfected into HEK293T cells. Cell lysates were immunoblotted with anti-HA antibodies (left panel). Bars on the right represent the normalized cleaved fraction as measured in Fig. 3(b). (c) HEK293T cells were transfected with MUC16-Cter (F114HA, lane-1), MUC4-Cter (FM4-150HA, lane-3) and a chimera of membrane-proximal 12 aa of MUC16-Cter inserted into the membrane proximal region of otherwise uncleavable MUC4-Cter (FM4-ECD-M16-12ECD-M4TMCT-HA, lane-2). Cell lysates were immunoblotted with indicated antibodies.

As shown earlier, 12 membrane proximal ectodomain residues appear to be critical for MUC16 cleavage (Fig. 1b and 1f lane 8). In an effort to identify the exact site of cleavage, we performed alanine scan mutagenesis of the 12 residues, however, to our surprise it did not affect MUC16 cleavage (Fig. 5a). Further, complete deletion of the 12 amino acids from F114HA only partially abrogated MUC16 cleavage (Fig. 5b, lanes 1 and 2) and insertion of the 12 amino acids into MUC4-Cter (F-M4-150-HA) at the junction of TM and ECD resulted in its partial cleavage (Fig. 5c) that otherwise does not undergo cleavage. Our findings suggest that the cleavage of MUC16 is not entirely dictated by its primary amino acid sequence.

### Nuclear localization of MUC16-Cter is independent of its putative nuclear localization signal

**Figure 6:**
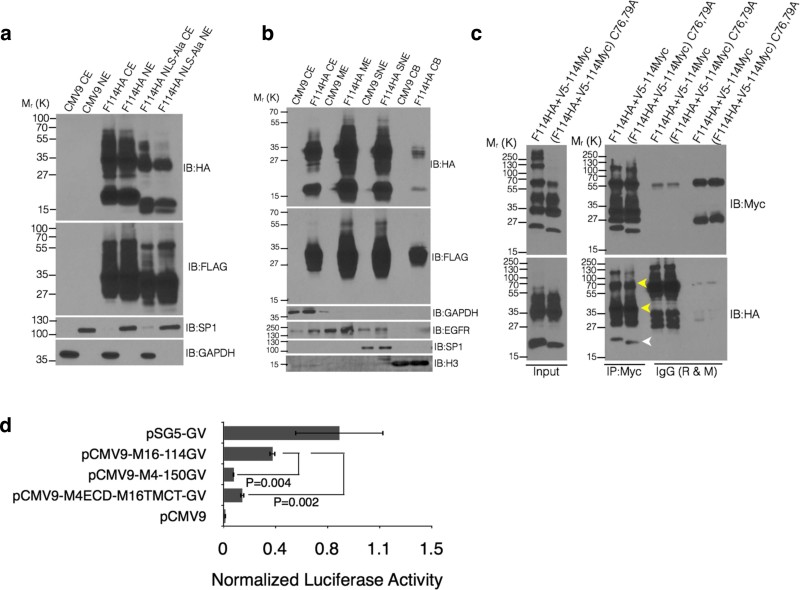
Cleavage dependent nuclear translocation and chromatin enrichment of MUC16-Cter. (a) Nuclear localization of MUC16-Cter is independent of its putative nuclear localization signal ‘RRRKKE’ (NLS). HEK293T cells transiently transfected with control (CMV9) or wild type (F114HA) or NLS-mutated versions of MUC16-Cter (F114HA NLS-Ala) were lysed and cytoplasmic (CE) and nuclear (NE) fractions were isolated. Western blots were performed on the subcellular fractions with the indicated antibodies. (b) MUC16-Cter is found in the chromatin-bound subcellular fraction. HEK293T cells transiently transfected with control (CMV9) or MUC16-Cter (F114HA) cells were lysed and cytoplasmic (CE), membrane (ME), soluble nuclear (SNE) and chromatin-bound (CB) were isolated. Western blots were performed on the subcellular fractions with the indicated antibodies. (c) MUC16-Cter interacts with itself in a disulfide linkage(s)-independent manner. HEK293 cells were cotransfected with CMV9-F114HA and pSecTag2C-V5-114Myc plasmids (N-ter V5-tag and C-ter Myc-tag) or Cys76/79Ala (C76,79A) versions of both. Lysates were immunoprecipitated using either control antibody (IgG, rabbit-R and mouse-M) or Myc-tag antibody and were immunoblotted with the indicated antibodies. Whole cell lysates from the same samples were used to detect the expression of WT and mutant (C76,79A) MUC16 C-ter using anti-HA and anti-Myc antibodies (Input). (d) Nuclear localization of MUC16-Cter is dependent on its ability to undergo cleavage. C-terminal fusions of GAL4-VP16 were generated for MUC16-114, MUC4-150 (pCMV9-M4-150GV) and MUC4-ECD-MUC16TMCT (pCMV9-M4ECD-M16TMCT-GV), of which the latter two have been shown not to undergo cleavage. (Fig. 1f, lane 3 and 6). Luciferase assay was carried out to assess the effect of cleavage in nuclear translocation. pSG5-GV was used as a positive control for the assay.

MUC16-Cter is predicted to have a nuclear localization signal (NLS i.e. RRRKKE) without DNA-binding domain and was detected in both soluble nuclear extract (SNE) and the chromatin bound (CB) fraction (Fig. 6a and 6b). However, mutation of the putative NLS (RRRKKE → AAAAAA; NLS → Ala) did not abrogate its nuclear localization (Fig. 6a), indicating involvement of a non-classical nuclear import pathway. The MUC1-Cter, despite having one RRK motif in the CTD, translocates into the nucleus by interacting with Nup62 independent of the RRK motif^38^. A prerequisite for this nuclear import is MUC1-Cter oligomerization^38^ mediated by disulfide bridges, the inhibition of which prevents Nup62 binding. To gain insight into the interaction of MUC16-Cter with itself, we investigated possible cysteine-mediated oligomerisation through expression of a pair of dual tagged MUC16-Cter constructs. Co-expression of FA114HA (N-ter FLAG and C-ter HA-tag) with V5-114MyC (N-ter V5-tag and C-ter Myc-tag) and co-immunoprecipitation showed self-interaction of MUC16-Cter. The results indicate possible heterodimerization between the cleaved fragments (white arrow head) as well as between cleaved and uncleaved MUC16-Cter fragments (yellow arrow heards) (Fig. 6c). Mutation of cysteine residues (C76,79A) did not affect the pattern or extent of MUC16-Cter self-interaction, suggesting strong non-covalent association resistant to denaturing conditions (Fig. 6c). Next, to understand if the cleavage of MUC16-Cter has any influence on nuclear translocation, chimeric constructs with C-terminal GAL4-VP16 (GV)^39^ were generated for MUC16-Cter (M16-114-GV), MUC4-Cter (M4-150-GV) and MUC4-ECD and MUC16-TMCT chimera (M4-ECD-M16TMCT-GV). There were significant reductions in the luciferase activities of M4-150-GV and M4-ECD-M16TMCT-GV, which unlike MUC16-Cter do not undergo cleavage (Fig. 6d), demonstrating the dependence of nuclear translocation on the cleavage.

## Discussion

CA125, since its discovery in 1981^40^, is used as the gold standard biomarker for ovarian cancer. Its molecular identity was revealed to be a transmembrane mucin, MUC16 in 2001^14^,^41^. Recent studies have further underscored its importance; owing to its *de novo* and/or increased expression in cancer^5^,^6^,^7^ as well as being one of the top three frequently mutated genes across various cancer types^42^. Given the importance of oncogenic signaling mediated by mucin cytoplasmic tail following cleavage^2^, we investigated the speculations about MUC16 cleavage^12^,^14^. Here, we demonstrate for the first time that the 12 extracellular amino acids proximal to the TM domain are sufficient for MUC16 cleavage opposed to the proposed 50 aa in the last SEA domain (site #1, PLARRVDR)^14^ and a second cleavage (site # 2) at ‘DSVLV’ site in the penultimate SEA domain^12^ analogous to ‘GSVVV’ of MUC1. Although we have not been able to demonstrate cleavage of endogenous MUC16 due to commercial unavailability of CTD specific antibodies, a recent study published by Davies *et al.*^43^, using an in-house antibody raised against the MUC16 CTD, demonstrates the existence of a ~17 kDa cleaved product in NHBE cells. This supports our findings in an overexpression system. Although we demonstrated that the membrane proximal 12 amino acids were sufficient for MUC16 cleavage, deletion of the same only partially abrogated its cleavage suggesting this stretch of 12 amino acids is not an absolute necessity for its cleavage. The possible reasons for this could be: (i) cleavage of MUC16 is not dictated by its primary amino acid sequence, instead by a change in its structure as it encounters the acidic pH of the secretory pathway, or (ii) existence of closely apposed multiple cleavage sites that are independent of each other. Therefore, our future work will be directed towards testing the above-mentioned hypotheses of MUC16 cleavage.

The protein backbone of MUC16 is ~22,152 amino acids, the largest among all the known mucins and corresponds to ~2.5 MDa unglycosylated and 20 MDa glycosylated mass^8^. While CA125 is considered to be the extracellular shed portion of MUC16 following cleavage, it is hypothesized to be a discontinuous repetitive epitope distributed across the tandem repeat region of MUC16^21^. Since the antibodies against CA125 detect a protein of approximately 200–250 kDa (i.e. 0.2–0.25 MDa), much smaller than the N-ter extracellular fragment of MUC16, our findings of the most distal cleavage of MUC16 at the juxta membrane ectodomain region do not necessarily generate CA125. Therefore, while the cleavage reported in here provides an explanation for the release of MUC16 from the cell surface, the mechanism(s) of CA125 generation still remains to be elucidated.

Since cleavage of MUC16 takes place in the membrane proximal ectodomain *en route* to the plasma membrane, use of antibodies targeting extracellular tandem repeat regions of MUC16 is not the most effective means of targeting cancer cells and is considered to be one of the major factors for the failures in clinical trials using this approach. In a recent study, Dharma Rao *et al*.^44^ have generated several monoclonal antibodies targeting the membrane proximal ectodomain and the CTD of MUC16. These have been shown to be effective in multiple applications and therefore are expected to be useful in diagnostics and therapeutics. However, the antibodies for the membrane proximal region are generated assuming cleavage of MUC16 at 50 residues upstream of the TM domain (site #1, PLARRVDR) and therefore does not bind to amino acids in the F65HA fragment that we demonstrate here to be the minimum length of MUC16 carboxyl-terminal fragment that undergoes cleavage. Therefore in addition to the above-mentioned antibodies, additional antibodies towards carboxyl-terminus MUC16 including the membrane proximal 12 residues will be critical for diagnostic and therapeutic targeting using MUC16.

We further demonstrated that the molecular weight disproportionate to the different lengths of polypeptides expressed is due to post-translational modifications (PTMs) such as N-glycosylation, ubiquitylation and/or self di-/oligomerization. These PTMs are probably critical for its subcellular localizations as well as biological functions as they regulate the stability of MUC16-Cter. Specifically, mutations of the N-glycosylation site(s) such as Asn30Gln and Asn24,30Gln abrogated the higher molecular weight glycoforms of F114HA. In addition, the intensity of 17 kDa cleaved MUC16 was reduced in the N-glycosylation mutants suggesting cleavage of MUC16 to be influenced at least in part by N-glycosylation. However, F65HA with no N-glycosylation sites was cleaved as efficiently as other longer fragments with different numbers of N-glycosylation sites. Therefore, while it appears that N-glycosylation influences MUC16 cleavage, further studies would be required to either implicate or rule out the involvement of N-glycosylation in MUC16 cleavage.

The juxtamembrane stretch of positively charged Lys/Arg residues (RRRKK), though was considered to be the putative nuclear localization signal, mutation of this stretch to Ala did not abrogate its ability to undergo nuclear localization, suggesting involvement of non-canonical nuclear transport. One possibility could be the di/oligomerization induced importin-β mediated nuclear transport as has been shown for MUC1-Cter^38^. However, the oligomerization of MUC1-Cter is mediated by the disulfide linkages mediated by Cys residues, which is not true for MUC16-Cter. Therefore, future studies should be directed towards understanding the mechanistic basis of di/oligomerization as well as nuclear translocation of MUC16-Cter. This suggests that MUC16-Cter could have regulatory effect on the gene expression possibly mediated by interaction with other transcription factor(s) and regulatory protein(s) in the nucleus. Indeed, we demonstrated that expression of MUC16-Cter in MiaPaCa-2 pancreatic cancer cells resulted in up regulation of *LMO2* and *NANOG*, implicated in inducing stem-cell like features during carcinogenesis, in a JAK2 dependent manner^24^. Further, our demonstration of the dependence of increased nuclear activity using GAL4-VP16 system on the ability of MUC16-Cter to undergo cleavage suggests that understanding the exact mechanism of cleavage would enable us to design specific therapeutic intervention(s) targeted to prevent cleavage of MUC16.

Understanding cleavage and post-cleavage events of MUC16 would aid in dissecting its role in multiple malignancies and its *de novo* expression in PC makes it a suitable candidate to be exploited for targeted therapy. Preventing cleavage of MUC16 would serve two important purposes such as (i) reduced nuclear translocation, and (ii) increased cell surface representation of MUC16 that will enhance the efficacy of the CA125 antibody based drugs such as Oregovomab and Abagovomab. Here, we showed that (i) increased nuclear translocation of the cleaved fragment is dependent of MUC16 cleavage, and (ii) treatment of BFA results in abrogation of MUC16 cleavage. Although use of drugs that perturbs the secretory pathway raises the obvious concern of affecting the functions of normal cells, studies have demonstrated that normal peripheral blood mononuclear cells, fibroblasts and retinal pigment epithelial cells are much less sensitive to BFA treatment compared to the malignant cells, possibly due to the increased reliance of the tumor cells on the secretory pathway compared to the normal cells^35^,^45^. Breflate (a prodrug form of brefeldin-A, NSC656202) can be used to prevent cleavage of MUC16 and its associated tumorigenic functions and therefore can be viewed as an interesting therapeutic avenue^35^.

## Methods

### Cell culture and transfections

HEK293T, HeLa, MiaPaCa-2, T3M4, MCF7 and SKOV3 cells were grown in DMEM supplemented with 10% heat-inactivated FBS (Sigma) and 100 U/ml penicillin, 100 μg/ml streptomycin (penstrep). HPDE was cultured in keratinocyte serum free medium supplemented with EGF, bovine pituitary extract and penstrep. MCF10-A cells were cultured in DMEM/F12 medium supplemented with 5% heat-inactivated FBS, EGF (20 μg/ml), hydrocortisone (0.5 μg/ml), cholera toxin (0.1 μg/ml), insulin (10 μg/ml) and penstrep. Parental (PRO^−5^) and N-glycosylation deficient (Lec-1 and Lec-8) CHO and U-937 cells (suspension culture) cells were grown in RPMI medium supplemented with 5% heat-inactivated FBS and penstrep. Transient transfections of HEK293T cells were performed using PEI (polyethyleneimine) at a ratio of 5 ug PEI/ug of DNA. MiaPaCa-2, T3M4, HPDE, MCF10-A, MCF7, SKOV3, HeLa and CHO cells were transfected using Lipofectamine 2000 (Invitrogen) according to the manufacturer's instructions. MiaPaCa-2 and T3M4 cells were selected for G418 (400–600 μg/ml) resistance to generate the stable cells. These stable cells were maintained in 400 μg/ml G418 only except during the experimental procedure.

### Plasmids and cloning strategy

Standard PCR and molecular cloning techniques were used to make constructs. For expression in the mammalian system, p3X-FLAG-CMV9 (Sigma) and pSecTag2C (Invitrogen) plasmids were used to make various constructs. DNA fragments encoding the carboxyl-terminal region of MUC16 (321 amino acids), MUC4 (150 amino acids), was amplified by RT-PCR and was cloned into the respective expression vectors. These constructs were further manipulated to generate various point mutations (by site-directed mutagenesis), deletions and domain swapping variants and are listed in Supplementary Table 1. Gal4-VP16 was PCR-amplified from pSG5-SP-C99-Gal4-VP16 plasmid and cloned C-terminal to MUC16-114, MUC4-150 and MUC4-ECD-MUC16TMCT in the p3X-FLAG-CMV9 vector.

### Establishment of skin fibroblasts from adult WT, Mmp7^−/−^ and Mmp2^−/−^ mice

Fibroblasts were prepared from the skin of wild type (C57BL/6), Mmp2^−/−^ and Mmp7^−/−^ KO mice as described previously^46^. Within 3–6 passages these fibroblasts were used for the purpose of transfection and RNA extraction. The Institutional Animal Care and User Committee at the University of Nebraska Medical Center approved all animal work.

### Immunoprecipitation and immunoblotting

Cells were lysed in IP buffer (50 mM Tris-HCl pH-7.4, 300 mM NaCl, 5 mM EDTA, 1% NP-40) containing complete protease inhibitor cocktail (Roche), 2 mM Na_3_VO_4_, 10 mM NaF and 1 mM PMSF on ice for 30 minutes. Cell lysates were clarified by centrifugation and were immunoprecipitaed with indicated antibodies overnight at 4°C. Protein complexes were isolated by incubation with Protein-A, Protein-G or Protein-A/G Agarose beads (Santa Cruz Biotechnology) for 2–4 h. Immunoprecipitates were washed 3–5 times with IP buffer, boiled with SDS sample buffer and analysed by immunoblotting as described below using indicated antibodies. Standard methods were used for immunoblotting. Cells were lysed with RIPA buffer (50 mM Tris-HCl pH-7.5, 150 mM NaCl, 1% NP-40, 0.5% sodium deoxycholate, and 0.1% SDS) supplemented with complete protease inhibitor mixture (Roche), 2 mM Na_3_VO_4_, 10 mM NaF and 1 mM PMSF on ice. Cell lysates were cleared by centrifugation and quantified using the bicinchoninic acid method. Proteins (10–40 μg) were separated by SDS–PAGE under reducing conditions and blotted onto a polyvinylidene difluoride membrane (Millipore). Membranes were probed with specific antibodies. Blots were washed and probed with respective secondary peroxidase-conjugated antibodies, and the bands visualized by chemiluminescence (Thermo Scientific). The following antibodies were used: mouse monoclonal antibodies for FLAG-Tag (1:3000; M2), β-Actin (1:5000) from Sigma, Myc-tag (1:2000) from Cell signaling, EGFR (1:1000; Santa Cruz Biotechnology), rabbit monoclonal antibodies for HA-Tag (1:2000), GAPDH (1:1000), SP1 (1:1000) from Cell Signaling, and rabbit polyclonal antibody for Histone H3 (1:1000; Abcam).

### In vivo ubiquitylation assay

HEK293T cells coexpressing HA-tagged wild type (F114HA) or lysine (F114HA-2K → A), cysteine (F114HA-3C → A), lysine and cysteine (F114HA-2K → A/3C → A), Lys89Ala (F114HA K89A), Lys90Ala (F114HA K90A) mutants of MUC16-Cter and Myc-tagged Ubiquitin were lysed with IP buffer (50 mM Tris-HCl pH-7.4, 300 mM NaCl, 5 mM EDTA, 1% NP-40) supplemented with complete protease inhibitor mixture (Roche), 2 mM Na_3_VO_4_, 10 mM NaF, 1 mM PMSF and placed on ice for 20 min. Cell lysates were cleared by centrifugation and immunoprecipitated using indicated antibodies for 2 h to overnight at 4°C. Protein complexes were collected by incubation for 2–4 h with Protein-A, Protein-G Agarose beads (Santa Cruz Biotechnology). Immunoprecipitates were washed 3–5 times with IP buffer, boiled in SDS sample buffer and analyzed by immunoblotting with appropriate antibodies.

### Subcellular fractionation

Subcellular fractionations were carried out using subcellular protein fractionation kits (Thermo Scientific and G-Biosciences) according to the manufacturers' instructions. Fraction purity was determined by western blotting, using the following antibodies: GAPDH for cytoplasmic, EGFR for membrane, SP1 for total and soluble nuclear, and Histone-3 for chromatin-bound fractions.

### Luciferase assay

0HEK293T cells were transfected with Renilla Luciferase plasmid (25 ng), pFR-Luciferase plasmid (500 ng) and the inducer plasmids (1000 ng) at a ratio of 1:20:40 respectively. Inducer plasmids included pCMV9-M16-114-GAL4-VP16, pCMV9-M4-150-GAL4-VP16, pCMV9-M4ECD-M16TMCT-GAL4-VP16, pSG5-APP-C99-GAL4-VP16, pSG5-GAL4-VP16 or pCMV9 empty vector. Luciferase readings were measured 24 h following transfection according to manufacturers' instructions (Promega) with the exception of Υ-secretase mediated RIP assay. For this, 24 h after transfection cells were treated with or without inhibitor X (25 nM Inh X; Calbiochem) for a period of 16 h before luciferase readings were measured.

### Immunofluorescence microscopy

HeLa cells were grown on coverslips for twenty-four hours and were washed twice with PBS and fixed with 4% paraformaldehyde in PBS (pH-7.4) for 10 min. After washing with PBS, cells were quenched with 30 mM glycine. Cells were then permeabilized with 0.1% Triton-X-100 for 10 min and blocked with 10% normal goat serum (NGS) in PBS for 1 h. Cells were incubated with appropriate antibodies (anti-Calnexin; 1:200 from SCB, anti-Giantin; 1:1000 from Abcam) for 1 h in PBS containing 2% NGS. The cells were washed three times with PBST and incubated with Alexa-Fluor 488-conjugated donkey anti-mouse and Alexa-Fluor 568-conjugated donkey anti-rabbit (Life Technologies) antibodies for 30 min. The cells were washed three times with PBST and mounted in Vectashield with DAPI (Vector Laboratories).

### RNA isolation, reverse transcription and PCR analysis

RNA was extracted from the cells using the RNeasy mini kit (Qiagen) according to the manufacturer's instructions. cDNA was synthesized from total RNA using oligo-dT or random hexamers using SuperScript reverse transcriptase II (Life Technologies) kit. For analysis of neutrophil elastase (*ELANE*), *MMP7*, *Mmp2* and *Mmp7*, gene specific primers were designed using NCBI primer designing tool and endpoint PCR was carried out for 30 cycles with a melting temperature of 58°C. β-*ACTIN* and *Gapdh* were used as loading controls.

Primers used are MMP-7 FP: 5′- CAGGAAACACGCTGGCTCAT-3′ RP: 5′-AGACTGCTACCATCCGTCCA-3′, ELANE FP: 5′-CATATAGATCTCATCTGGGCATCC-3′ RP: 5′-TGCCAGATGCTGGAGAGTGT-3′, Mmp-7 FP: 5′-TGGAGACAGCTTCCCCTTTG-3′ RP: 5′- TGGAGACAGCTTCCCCTTTG-3′, Mmp-2 FP: 5′- TCCCCCGATGCTGATACTGA-3′ Mmp-2 RP: 5′- TCCCCCGATGCTGATACTGA-3′, β-ACTIN FP: 5′-TGGACATCCGCAAAGACCTG-3′ RP: 5′-TGGACATCCGCAAAGACCTG-3′, Gapdh FP: 5′-GCTCACTGGCATGGCCTTCCGTG-3′ RP: 5′-TGGAAGAGTGGGAGTTGCTGTTGA-3′.

### Cleaved fraction calculation

Since MUC16-Cter cleavage does not proceed to completion (Fig. 1b and 3b) and the cleavage takes place in the Golgi/post-Golgi compartments (Fig. 4b), measuring the FLAG-tagged product in the culture medium (assuming it is released) may not be a true measure of cleavage. To estimate the cleavage efficiency, we first normalized the bottom (<17 kDa C-ter HA-tagged product that is physically separated from the N-ter) and top (higher molecular weight) HA-tagged products with β-actin separately. A ratio of the normalized bottom to the total (bottom + top) HA-tagged fraction was calculated and was considered to be a better measure of cleavage efficiency, assuming the top HA-tagged to be either uncleaved and/or heterodimeric and the bottom HA-tagged to be the actual cleaved fraction (Fig. 3b and 5b, right panel). We employed this quantification to demonstrate no influence of the cytoplasmic tail amino acid mutations on MUC16-Cter cleavage (Fig. 3b, right panel). Though the mutations such as F114HA Tyr94/103/104Ala (3Y → A) appear to have an increased amount of 17 kDa cleaved fragment, the normalized cleaved fraction is unaltered compared to the WT (Fig. 5b, right panel), indicating these mutations may affect stability but not cleavage of MUC16-Cter.

## Supplementary Material

Supplementary Information Supplementary Information

## References

[b1] Hollingsworth M. A. & Swanson B. J. Mucins in cancer: protection and control of the cell surface. Nat. Rev. Cancer 4, 45–60 (2004). 14681689 10.1038/nrc1251

[b2] Kufe D. W. Mucins in cancer: function, prognosis and therapy. Nat. Rev. Cancer 9, 874–885 (2009). 19935676 10.1038/nrc2761 PMC2951677

[b3] Senapati S., Das S. & Batra S. K. Mucin-interacting proteins: from function to therapeutics. Trends Biochem. Sci. 35, 236–245 (2010). 19913432 10.1016/j.tibs.2009.10.003 PMC3030310

[b4] Mai P. L., Wentzensen N. & Greene M. H. Challenges related to developing serum-based biomarkers for early ovarian cancer detection. Cancer. Prev. Res. (Phila) 4, 303–306 (2011). 21372029 10.1158/1940-6207.CAPR-11-0053 PMC3077065

[b5] Einama T. *et al.* Co-expression of mesothelin and CA125 correlates with unfavorable patient outcome in pancreatic ductal adenocarcinoma. Pancreas 40, 1276–1282 (2011). 21775916 10.1097/MPA.0b013e318221bed8

[b6] Streppel M. M. *et al.* Mucin 16 (cancer antigen 125) expression in human tissues and cell lines and correlation with clinical outcome in adenocarcinomas of the pancreas, esophagus, stomach, and colon. Hum. Pathol. 43, 1755–1763 (2012). 22542127 10.1016/j.humpath.2012.01.005 PMC3547617

[b7] Shimizu A. *et al.* Coexpression of MUC16 and mesothelin is related to the invasion process in pancreatic ductal adenocarcinoma. Cancer. Sci. 103, 739–746 (2012). 22320398 10.1111/j.1349-7006.2012.02214.x PMC7659350

[b8] Hattrup C. L. & Gendler S. J. Structure and function of the cell surface (tethered) mucins. Annu. Rev. Physiol. 70, 431–457 (2008). 17850209 10.1146/annurev.physiol.70.113006.100659

[b9] Govindarajan B. & Gipson I. K. Membrane-tethered mucins have multiple functions on the ocular surface. Exp. Eye Res. 90, 655–663 (2010). 20223235 10.1016/j.exer.2010.02.014 PMC2893012

[b10] Duraisamy S., Ramasamy S., Kharbanda S. & Kufe D. Distinct evolution of the human carcinoma-associated transmembrane mucins, MUC1, MUC4 AND MUC16. Gene 373, 28–34 (2006). 16500040 10.1016/j.gene.2005.12.021

[b11] Wreschner D. H. *et al.* Generation of ligand-receptor alliances by “SEA” module-mediated cleavage of membrane-associated mucin proteins. Protein Sci. 11, 698–706 (2002). 11847293 10.1110/ps.16502 PMC2373471

[b12] Macao B., Johansson D. G., Hansson G. C. & Hard T. Autoproteolysis coupled to protein folding in the SEA domain of the membrane-bound MUC1 mucin. Nat. Struct. Mol. Biol. 13, 71–76 (2006). 16369486 10.1038/nsmb1035

[b13] Akhavan A., Crivelli S. N., Singh M., Lingappa V. R. & Muschler J. L. SEA domain proteolysis determines the functional composition of dystroglycan. FASEB J. 22, 612–621 (2008). 17905726 10.1096/fj.07-8354com

[b14] O'Brien T. J. *et al.* The CA 125 gene: an extracellular superstructure dominated by repeat sequences. Tumour Biol. 22, 348–366 (2001). 11786729 10.1159/000050638

[b15] Theriault C. *et al.* MUC16 (CA125) regulates epithelial ovarian cancer cell growth, tumorigenesis and metastasis. Gynecol. Oncol. 121, 434–443 (2011). 21421261 10.1016/j.ygyno.2011.02.020

[b16] Boivin M., Lane D., Piche A. & Rancourt C. CA125 (MUC16) tumor antigen selectively modulates the sensitivity of ovarian cancer cells to genotoxic drug-induced apoptosis. Gynecol. Oncol. 115, 407–413 (2009). 19747716 10.1016/j.ygyno.2009.08.007

[b17] Akita K. *et al.* CA125/MUC16 interacts with Src family kinases, and over-expression of its C-terminal fragment in human epithelial cancer cells reduces cell-cell adhesion. Eur. J. Cell Biol. 92, 257–263 (2013). 24246580 10.1016/j.ejcb.2013.10.005

[b18] Matte I., Lane D., Boivin M., Rancourt C. & Piche A. MUC16 mucin (CA125) attenuates TRAIL-induced apoptosis by decreasing TRAIL receptor R2 expression and increasing c-FLIP expression. BMC Cancer 14, 234-2407-14-234 (2014). 10.1186/1471-2407-14-234 PMC4234371 24690311

[b19] Blalock T. D., Spurr-Michaud S. J., Tisdale A. S. & Gipson I. K. Release of membrane-associated mucins from ocular surface epithelia. Invest. Ophthalmol. Vis. Sci. 49, 1864–1871 (2008). 18436821 10.1167/iovs.07-1081 PMC2622730

[b20] Govindarajan B. *et al.* A metalloproteinase secreted by Streptococcus pneumoniae removes membrane mucin MUC16 from the epithelial glycocalyx barrier. PLoS One 7, e32418 (2012). 22412870 10.1371/journal.pone.0032418 PMC3296694

[b21] Felder M. *et al.* MUC16 (CA125): tumor biomarker to cancer therapy, a work in progress. Mol. Cancer 13, 129-4598-13-129 (2014). 10.1186/1476-4598-13-129 PMC4046138 24886523

[b22] Berek J. S. *et al.* Randomized, placebo-controlled study of oregovomab for consolidation of clinical remission in patients with advanced ovarian cancer. J. Clin. Oncol. 22, 3507–3516 (2004). 15337799 10.1200/JCO.2004.09.016

[b23] Sabbatini P. *et al.* Abagovomab as maintenance therapy in patients with epithelial ovarian cancer: a phase III trial of the AGO OVAR, COGI, GINECO, and GEICO--the MIMOSA study. J. Clin. Oncol. 31, 1554–1561 (2013). 23478059 10.1200/JCO.2012.46.4057 PMC5795662

[b24] Das S. *et al.* Carboxyl-terminal domain of MUC16 imparts tumorigenic and metastatic functions through nuclear translocation of JAK2 to pancreatic cancer cells. Oncotarget (2015). 10.18632/oncotarget.3308 PMC4467401 25691062

[b25] Patnaik S. K. & Stanley P. Lectin-resistant CHO glycosylation mutants. Methods Enzymol. 416, 159–182 (2006). 17113866 10.1016/S0076-6879(06)16011-5

[b26] Mumm J. S. *et al.* A ligand-induced extracellular cleavage regulates gamma-secretase-like proteolytic activation of Notch1. Mol. Cell 5, 197–206 (2000). 10882062 10.1016/s1097-2765(00)80416-5

[b27] Sisodia S. S. & St George-Hyslop P. H. gamma-Secretase, Notch, Abeta and Alzheimer's disease: where do the presenilins fit in? Nat. Rev. Neurosci. 3, 281–290 (2002). 11967558 10.1038/nrn785

[b28] Xiong L., Woodward A. M. & Argueso P. Notch signaling modulates MUC16 biosynthesis in an in vitro model of human corneal and conjunctival epithelial cell differentiation. Invest. Ophthalmol. Vis. Sci. 52, 5641–5646 (2011). 21508102 10.1167/iovs.11-7196 PMC3176047

[b29] Tousseyn T. *et al.* ADAM10, the rate-limiting protease of regulated intramembrane proteolysis of Notch and other proteins, is processed by ADAMS-9, ADAMS-15, and the gamma-secretase. J. Biol. Chem. 284, 11738–11747 (2009). 19213735 10.1074/jbc.M805894200 PMC2670177

[b30] Urban S. Making the cut: central roles of intramembrane proteolysis in pathogenic microorganisms. Nat. Rev. Microbiol. 7, 411–423 (2009). 19421188 10.1038/nrmicro2130 PMC2818034

[b31] Rawson R. B. *et al.* Complementation cloning of S2P, a gene encoding a putative metalloprotease required for intramembrane cleavage of SREBPs. Mol. Cell 1, 47–57 (1997). 9659902 10.1016/s1097-2765(00)80006-4

[b32] Fendrick J. L. *et al.* CA125 phosphorylation is associated with its secretion from the WISH human amnion cell line. Tumour Biol. 18, 278–289 (1997). 9276028 10.1159/000218041

[b33] Konishi I., Fendrick J. L., Parmley T. H., Quirk J. G. Jr & O'Brien T. J. Epidermal growth factor enhances secretion of the ovarian tumor-associated cancer antigen CA125 from the human amnion WISH cell line. J. Soc. Gynecol. Investig. 1, 89–96 (1994). 10.1177/107155769400100118 9419754

[b34] Colanzi A. *et al.* Molecular mechanism and functional role of brefeldin A-mediated ADP-ribosylation of CtBP1/BARS. Proc. Natl. Acad. Sci. U. S. A. 110, 9794–9799 (2013). 23716697 10.1073/pnas.1222413110 PMC3683763

[b35] Carew J. S. Targeting endoplasmic reticulum protein transport: a novel strategy to kill malignant B cells and overcome fludarabine resistance in CLL. Blood 107, 222; 222–231; 31 (2006). 10.1182/blood-2005-05-1923 PMC1895341 16144803

[b36] Nakamura N., Tanaka S., Teko Y., Mitsui K. & Kanazawa H. Four Na+/H+ exchanger isoforms are distributed to Golgi and post-Golgi compartments and are involved in organelle pH regulation. J. Biol. Chem. 280, 1561–1572 (2005). 15522866 10.1074/jbc.M410041200

[b37] Axelsson M. A. *et al.* Neutralization of pH in the Golgi apparatus causes redistribution of glycosyltransferases and changes in the O-glycosylation of mucins. Glycobiology 11, 633–644 (2001). 11479274 10.1093/glycob/11.8.633

[b38] Leng Y. *et al.* Nuclear import of the MUC1-C oncoprotein is mediated by nucleoporin Nup62. J. Biol. Chem. 282, 19321–19330 (2007). 17500061 10.1074/jbc.M703222200

[b39] Chapin H. C., Rajendran V., Capasso A. & Caplan M. J. Detecting the surface localization and cytoplasmic cleavage of membrane-bound proteins. Methods Cell Biol. 94, 223–239 (2009). 20362093 10.1016/S0091-679X(08)94011-5 PMC3063071

[b40] Bast R. C. Jr *et al.* Reactivity of a monoclonal antibody with human ovarian carcinoma. J. Clin. Invest. 68, 1331–1337 (1981). 7028788 10.1172/JCI110380 PMC370929

[b41] O'Brien T. J., Beard J. B., Underwood L. J. & Shigemasa K. The CA 125 gene: a newly discovered extension of the glycosylated N-terminal domain doubles the size of this extracellular superstructure. Tumour Biol. 23, 154–169 (2002). 12218296 10.1159/000064032

[b42] Kim N., Hong Y., Kwon D. & Yoon S. Somatic mutaome profile in human cancer tissues. Genomics Inform. 11, 239–244 (2013). 24465236 10.5808/GI.2013.11.4.239 PMC3897852

[b43] Davies J. R., Kirkham S., Svitacheva N., Thornton D. J. & Carlstedt I. MUC16 is produced in tracheal surface epithelium and submucosal glands and is present in secretions from normal human airway and cultured bronchial epithelial cells. Int. J. Biochem. Cell Biol. 39, 1943–1954 (2007). 17604678 10.1016/j.biocel.2007.05.013

[b44] Dharma Rao T. *et al.* Novel monoclonal antibodies against the proximal (carboxy-terminal) portions of MUC16. Appl. Immunohistochem. Mol. Morphol. 18, 462–472 (2010). 20453816 10.1097/PAI.0b013e3181dbfcd2 PMC4388147

[b45] Larsson D. E. *et al.* Identification and evaluation of potential anti-cancer drugs on human neuroendocrine tumor cell lines. Anticancer Res. 26, 4125–4129 (2006). 17201123

[b46] Seluanov A., Vaidya A. & Gorbunova V. Establishing primary adult fibroblast cultures from rodents. J. Vis. Exp. 44, e2033 (2010). 10.3791/2033 PMC3185624 20972406

