# Supplementary material for: Membrane proximal ectodomain cleavage of MUC16 occurs in the acidifying Golgi/post-Golgi compartments
Source: Sci Rep. 2015 Jun 5;5:9759. doi: 10.1038/srep09759 (PMC4456727; doi:10.1038/srep09759)
Supplement: Supplementary Information [file srep09759-s1.pdf]

**Supplementary Information**

# Membrane proximal ectodomain cleavage of MUC16 occurs in the acidifying Golgi/post-Golgi compartments

Srustidhar Das, Prabin D. Majhi, Mona H. Al-Mugotir, Satyanarayana Rachagani, Paul Sorgen, Surinder K. Batra

## Supplementary Figure Legends

**Supplementary Figure 1. Schematic representation of full-length and the 114 amino acid C-terminal fragment of MUC16.** (Top panel) Full-length MUC16 is comprised of ~ 22,152 amino acids with a huge N-terminal glycosylated region that habors a 12,000 amino acids long O-glycosylated region, 60+ tandem repeats of 156 amino acids each, a unique C-terminal region, a transmembrane domain and a short cytoplasmic tail. The predicted cleavage sites of MUC16 are noted as site #1 in the last ($56^{th}$) and site #2 in the penultimate ($55^{th}$) SEA domains. (Bottom Panel) Amino acid sequence of the C-terminal 114 residues ($22{,}038^{th}$ – $22{,}152^{nd}$ residues) of MUC16 from the predicted cleavage site #1. The amino acids with the numbers are noted in the sequence used for multiple site-directed mutagenesis used in this study.

**Supplementary Figure 2. Similar pattern of membrane proximal cleavage of MUC16 takes place in most cell types.** F114HA plasmid was transfected into non-tumorigenic (HPDE and MCF10A) or tumorigenic (MiaPaCa-2, T3M4, MCF7, SKOV3, HeLa, HEK293T) cell lines derived from various tissues (mentioned at the bottom of the immunoblot). Cell lysates were immunoblotted with anti-HA antibody.

Supplementary Figure 1

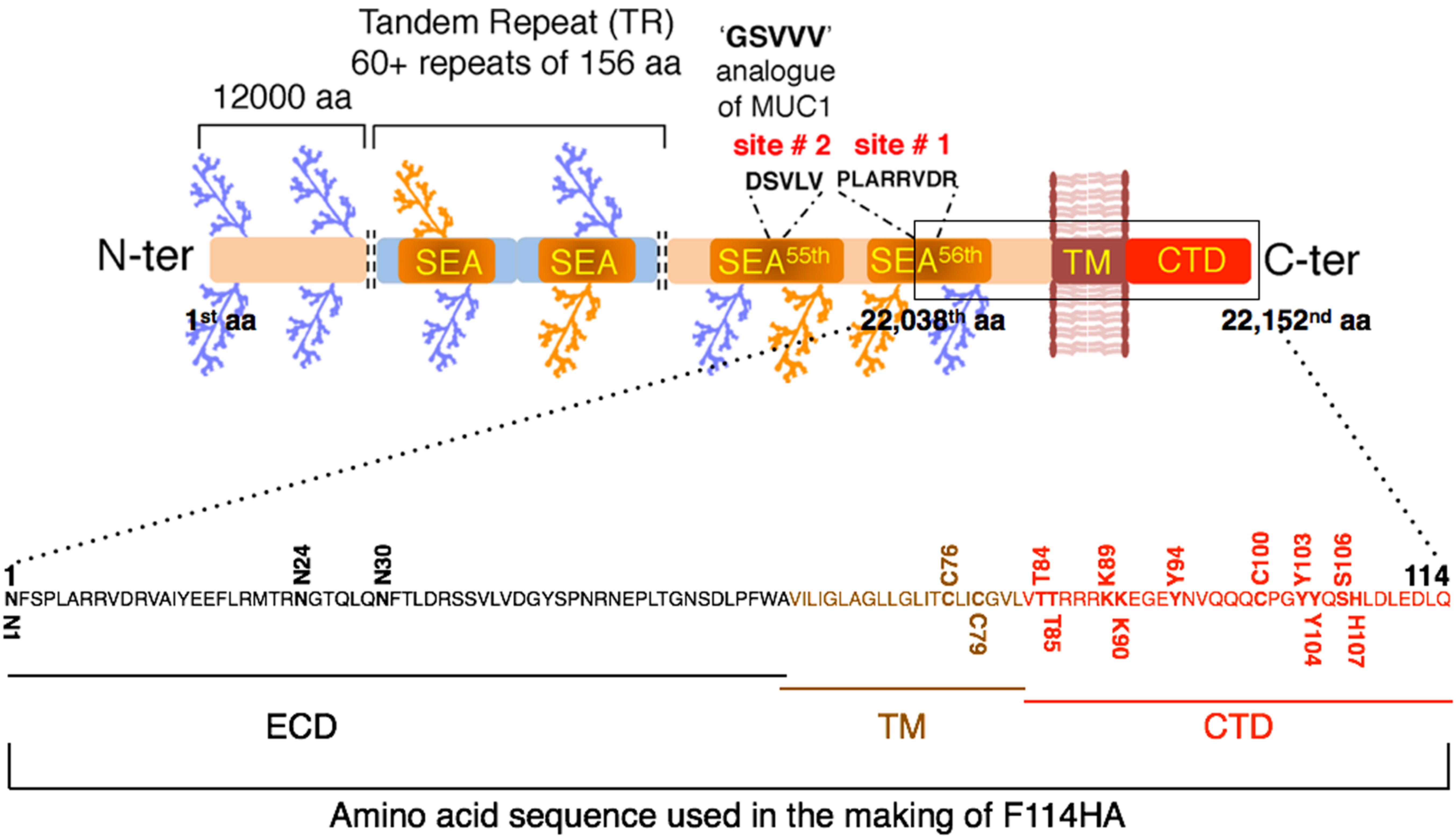

# Supplementary Figure 2

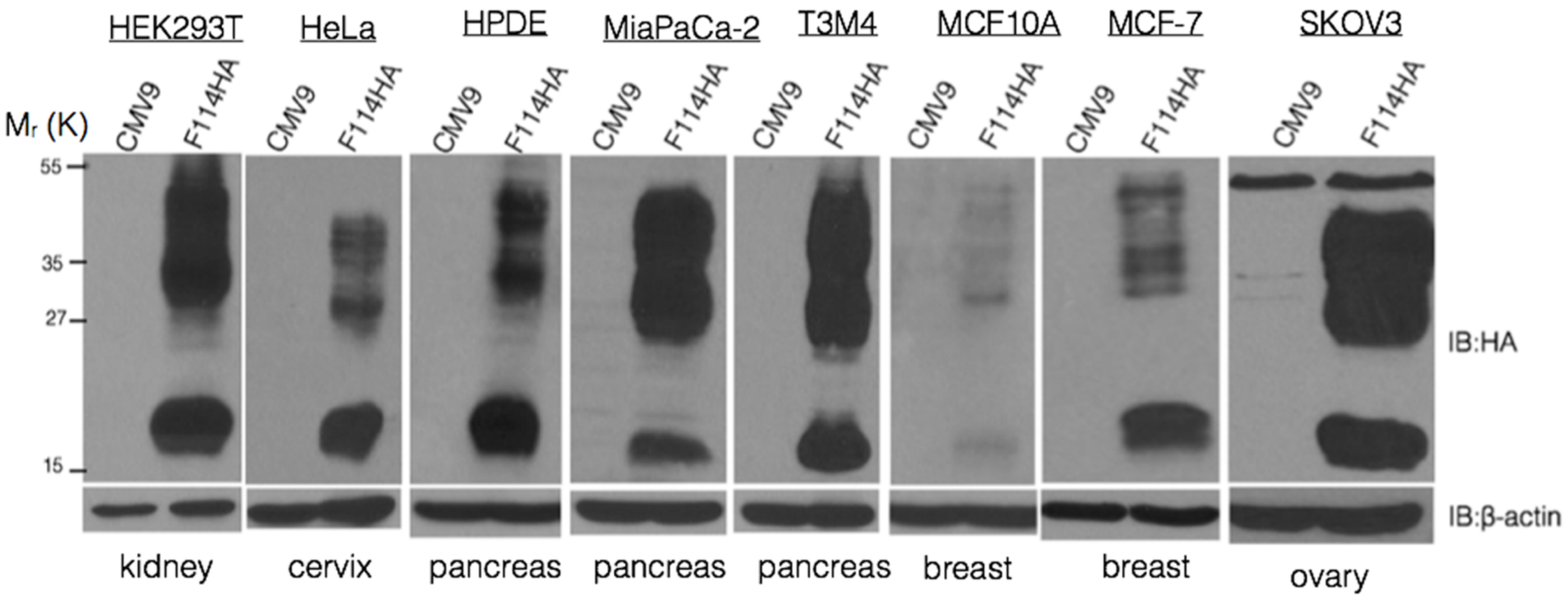

**Supplementary Table. 1 List of constructs used in the study (except point mutants)**

| SL No | Plasmid Name | Forward Primer | Reverse Primer |
|---|---|---|---|
| 1 | CMV9-F321HA | 5'–TACGAGATCTCTTCATCAATGGCTATGCACCCCAGAATTTATCAATCCGGGGCGAGTACCAGATAAATTTCCACATTGTCAACTGGAAC–3' | 5'–TACGGGATCCTCAAGCGTAATCTGGAACATCGTATGGGTAACCACCTTGCAGATCCTCCAGGTCTAGGT–3' |
| 2 | CMV9-F198HA | 5'–TACGAGATCTATCAGTTTATCAACCAACAAGCAGCTCCAGCACCCAGCACTTCTACCTGAATTTCACCATCACCAACCTAC–3' | 5'–TACGGGATCCTCAAGCGTAATCTGGAACATCGTATGGGTAACCACCTTGCAGATCCTCCAGGTCTAGGTG–3' |
| 3 | CMV9-F114HA | 5'–CTGAAGATCTAACTTCTCGCCACTGGCTCGGAGAG–3' | 5'–TACGGGATCCTCAAGCGTAATCTGGAACATCGTATGGGTAACCACCTTGCAGATCCTCCAGGTCTAGGTG–3' |
| 4 | CMV9-F65HA | 5'–TACGAGATCTGCCCTTAACTGGGAATTCTGACCTTCCCTTCTGGGCTGTCATCCTCATCG–3' | 5'–TACGGGATCCTCAAGCGTAATCTGGAACATCGTATGGGTAACCACCTTGCAGATCCTCCAGGTCTAGGTG–3' |
| 5 | CMV9-F53HA | 5'–TACGAGATCTAGTCATCCTCATCGGCTTGGCAGGACTCCTGGGAGTCATCACATGCCTGATCTGCGGTGTC–3' | 5'–TACGGGATCCTCAAGCGTAATCTGGAACATCGTATGGGTAACCACCTTGCAGATCCTCCAGGTCTAGGTG–3' |
| 6 | pST-HA321Myc | 5'–CTTAAAGCTTTATACCCATACGATGTTCCAGATTACGCTTTCATCAATGGCTATGCAC–3' | 5'–TACGCTCGAGTTGCAGATCCTCCAGGTCTAGGTGTGACTGGTAGTAGCCTGGGCAC–3' |
| 7 | pST-V5-114Myc | 5'–GTACAAGCTTTAGGTAAGCCTATCCCTAACCCTCTCCTCGGTCTCGATTCTACGAACTTCTCGCCACTGGCT–3' | 5'–TACGCTCGAGTTGCAGATCCTCCAGGTCTAGGTGTGACTGGTAGTAGCCTGGGCAC–3' |
| 8 | pST-HA1-239-FLAG-240-321Myc | 5'–CTTAAAGCTTTATACCCATACGATGTTCCAGATTACGCTTTCATCAATGGCTATGCAC–3'<br>5'–CTAGGATATCGATTACAAGGATGACGATGACAAGCTGGACAGGAGCAGTGTC–3' | 5'–GTACGATATCACCCTGAAAATACAAATTCTCGGTGAAGTTCTGCAGCTGG–3'<br>5'–TACGCTCGAGTTGCAGATCCTCCAGGTCTAGGTGTGACTGGTAGTAGCCTGGGCAC–3' |
| 9 | CMV9-F-M16(ECD-TM)+M4CTD-HA | 5'–CTGAAGATCTTAACTTCTCGCCACTGGCTCGGAG–3'<br>5'–ATGAGATATCGGTTGCTCCGGGGCCAGGTTCTC–3' | 5'–CTGAGATATCCCGCCGGCGGGTGGTCAC–3'<br>5'–ATGAGGATCCTCAAGCGTAATCTGGAACATCGTATGGGTAACCACCAGGCAAGGCCTC–3' |
| 10 | CMV9-F-M16ECD+M4(TM-CTD)-HA | 5'–CTGAAGATCTTAACTTCTCGCCACTGGCTCGGAG–3'<br>5'–CTGAGATATCATCTTCTTTGGGGCCCTGGGC–3' | 5'–GTATGATATCCCAGAAGGGAAGGTCAGAATTCCCAG–3'<br>5'–ATGAGGATCCTCAAGCGTAATCTGGAACATCGTATGGGTAACCACCAGGCAAGGCCTC–3' |
| 11 | CMV9-F-M4ECD+M16(TMCTD)-HA | 5'–CTAGAGATCTGAACGACGTGGTCTTCCAGCCCATTTCC–3'<br>5'–GTATGATATCGTCATCCTCATCGGCTTGGCAGGACTCCTGGG–3' | 5'–CTATGATATCGCCGAAGAACGCGTCGAGTTTCATGCTCAGGTGC–3'<br>5'–TACGGGATCCTCAAGCGTAATCTGGAACATCGTATGGGTAACCACCTTGCAGATCCTCCAGGTCTAGGTG–3' |
| 12 | CMV9-F-M16ECD+M4TM+M16CTD-HA | 5'–GTTAAAGCTTAACTTCTCGCCACTGGCTCGGAGAGTAGACAGAGTTG–3'<br>5'–GCCTAAGATCTATCTTCTTTGGGGCCCTGGGCG–3'<br>5'–GATTGATATCGTGACCACCCGCCGGCGGAAGAAGGAAG–3' | 5'–GTACAGATCTCCAGAAGGGAAGGTCAGAATTCCCAGTTAAGGGCTCATTTCTG–3'<br>5'–CGTAGATATCCCAGAAGCGCAGGACCACGAACGTCC–3'<br>5'–TACGGGATCCTCAAGCGTAATCTGGAACATCGTATGGGTAACCACCTTGCAGATCCTCCAGGTCTAGGTG–3' |
| 13 | CMV9-F-M4ECD+M16(CTD-TM+6ECD)-HA | 5'–CTAGAGATCTGAACGACGTGGTCTTCCAGCCCATTTCC–3'<br>5'–CTTATGATATCGACCTTCCCTTCTGGGCTGTCATCCTCATCGGC–3' | 5'–CTATGATATCGCCGAAGAACGCGTCGAGTTTCATGCTCAGGTGC–3'<br>5'–TACGGGATCCTCAAGCGTAATCTGGAACATCGTATGGGTAACCACCTTGCAGATCCTCCAGGTCTAGGTG–3' |

| | | | |
|---|---|---|---|
| 14 | CMV9-F-M4ECD+M16(CTD-TM+12ECD)-HA | 5'–CTAGAGATCTGAACGACGTGGTCTTCCAGCCCATTTCC–3'<br>5'–CTTATGATATCCCCTTAACTGGGAATTCTGACCTTCCCTTCTG–3' | 5'–CTATGATATCGCCGAAGAACGCGTCGAGTTTCATGCTCAGGTGC–3'<br>5'–TACGGGATCCTCAAGCGTAATCTGGAACATCGTATGGGTAACCACCTTGCAGATCCTCCAGGTCTAGGTG–3' |
| 15 | CMV9-F-M4-150HA | 5'–CTAGAGATCTGAACGACGTGGTCTTCCAGCCCATTTCC–3' | 5'–ATGAGGATCCTCAAGCGTAATCTGGAACATCGTATGGGTAACCACCAGGCAAGGCCTC–3' |
| 16 | CMV9-M16-114-Gal4-VP16 | 5'–CTGAAGATCTAACTTCTCGCCACTGGCTCGGAGAG–3'<br>5'–CGTGGATATCATGAAGCTACTGTCTTCTATCGAACAAGC–3' | 5'–ATGCGATATCTTGCAGATCCTCCAGGTCTAGGTGTGACTGGTAGTA–3'<br>5'–CGTAGGATCCTCAACCGTACTCGTCAATTCCAAGGGC–3' |
| 17 | CMV9-M4-150-Gal4-VP16 | 5'–CTAGAGATCTGAACGACGTGGTCTTCCAGCCCATTTCC–3'<br>5'–CGTGGATATCATGAAGCTACTGTCTTCTATCGAACAAGC–3' | 5'–CTTATAGTACTAGGCAAGGCCTCAGCTGAGTTC–3'<br>5'–CGTAGGATCCTCAACCGTACTCGTCAATTCCAAGGGC–3' |
| 18 | CMV9-M4ECD-M16TMCT-Gal4-VP16 | 5'–CTAGAGATCTGAACGACGTGGTCTTCCAGCCCATTTCC–3'<br>5'–CGTGGATATCATGAAGCTACTGTCTTCTATCGAACAAGC–3' | 5'–GTCATAGTACTTTGCAGATCCTCCAGGTCTAGG–3'<br>5'–CGTAGGATCCTCAACCGTACTCGTCAATTCCAAGGGC–3' |
| 19 | CMV9-F-M4ECD-M16-12ECD-M4TMCT-HA | 5'–CTAGAGATCTGAACGACGTGGTCTTCCAGCCCATTTCC–3'<br>5'–GTATGATATCCCCTTAACTGGGAATTCTGACCTTCCCTTCTGGGCTATCTTCTTTGGGG–3' | 5'–CTATGATATCGCCGAAGAACGCGTCGAGTTTCATGCTCAGGTGC–3'<br>5'–ATGAGGATCCTCAAGCGTAATCTGGAACATCGTATGGGTAACCACCAGGCAAGGCCTC–3' |
| 20 | CMV9-F114HAΔ53-65 | 5'–CTCCCAACAGAAATGAGGTCATCCTCATCGGCTT–3' | 5'–AAGCCGATGAGGATGACCTCATTTCTGTTGGGAG–3' |
| Tag code colors: HA-Tag, FLAG-Tag, V5-Tag CMV9=p3XFLAG-CMV9 vector, pST=pSecTag2C vector | | | |
